# Effects of antiplatelet, anticoagulant, and nonsteroidal anti-inflammatory drugs on upper gastrointestinal bleeding in geriatric patients

**DOI:** 10.1097/MD.0000000000042170

**Published:** 2025-05-30

**Authors:** Soner Onem, İbrahim Ethem Güven, Süleyman Dolu, Muge Gul Gulecoglu Onem, Taner Akyol

**Affiliations:** a Department of Gastroenterology, Samsun Education and Research Hospital, Samsun, Turkey; b Department of Gastroenterology, Yenimahalle Education and Research Hospital, Ankara, Turkey; c Department of Gastroenterology, Faculty of Medicine, Dokuz Eylul University, Izmir, Turkey; d Department of Medical Biochemistry, Samsun Training and Research Hospital, Samsun, Turkey.

**Keywords:** antithrombotic, gastrointestinal bleeding, geriatrics, nonsteroidal anti-inflammatory drugs

## Abstract

In patients presenting with upper gastrointestinal bleeding (UGIB), it is crucial to assess the risk factors associated with poor clinical outcomes. The aim of this study is to evaluate the impact of antiplatelet, anticoagulant, and nonsteroidal anti-inflammatory drug (NSAID) usage on clinical outcomes in geriatric patients with UGIB. This study was conducted between January 2022 and December 2023, retrospectively. In total, 297 patients presenting with UGIB who were over 65 years of age were included in the study. Patients were divided into 4 groups based on their medication: antiplatelet, anticoagulant, NSAID groups, and no drug. The need for blood transfusion, need for endoscopic treatment, rebleeding rate, need for surgery or interventional radiology, length of hospital stay, and 30-day mortality rate were compared between the groups. In total, 103 (34.6%) patients were using antiplatelets, 83 (27.9%) patients were using anticoagulants, 32 (10.7%) patients were using NSAIDs, and 79 (26.5%) patients were using none of them. Hypertension, diabetus mellitus, cardiovascular and cerebrovascular diseases were more common in patients with antiplatelet and anticoagulant groups. On admission in the anticoagulant group, Glasgow–Blatchford score was found to be higher, systolic blood pressure and hemoglobin levels were found to be lower. However, there was no significant difference between the 4 groups in terms of 30-day mortality, rebleeding, number of blood transfusion or length of hospital stay (*P* > .05 for all parameters). NSAID or antithrombotic use was not associated with poor outcomes in geriatric patients with UGIB.

## 1. Introduction

Acute upper gastrointestinal bleeding (UGIB) is the most frequently consulted indication to gastroenterologists among patients entering the emergency department.^[1,2]^ Despite technological advances and increased awareness of gastrointestinal bleeding, the mortality rate secondary to UGIB can be up to approximately 10%, and it is also associated with high morbidity.^[3]^ Therefore, it is mandatory to determine the risk factors associated with UGIB and evaluate the effects of these factors on clinical outcomes.

The geriatric patient population constitutes a large proportion of patients presenting to the emergency department with UGIB. Since clinical outcomes secondary to UGIB are associated with poorer outcomes in the geriatric patient population, the management of these patients is critical.^[4]^ In this context, the most commonly encountered risk factor in clinical practice is antithrombotic/nonsteroidal anti-inflammatory drug (NSAID) usage, due to the increased frequency of use of these medications in geriatric patients.^[5]^ Data in the literature regarding the effects of antithrombotic/NSAID use on UGIB outcomes reveal conflicting results.^[6–8]^ In addition, studies evaluating the effects of these drug groups on clinical outcomes in the geriatric patient population are insufficient. Therefore, literature data on this concept are lacking, and further studies are warranted, since they may carry significant clinical implications when left unaddressed.

In this study, our aim was to investigate the effect of antithrombotic and/or NSAID use on clinical outcomes in geriatric patients admitted with UGIB.

## 2. Materials and methods

Geriatric patients (age > 65) who were admitted to the emergency department with symptoms and signs of UGIB and underwent esophagogastroduodenoscopy (EGD) at our tertiary referral center between January 01, 2022 and December 31, 2023 were enrolled in the study. Patients with missing data, those who presented with variceal bleeding, or patients who could not undergo EGD were excluded from the study. Patients who developed gastrointestinal bleeding during hospitalization were also excluded from the study. Patients who were prescribed multiple classes of medications in the NSAID, antiplatelet, and anticoagulant groups were excluded from the study. This study was approved by the Samsun University Clinical Research Ethics Committee (protocol number 2024/2/1, date January 17, 2024).

Demographic data, medication history, comorbidities (atrial fibrillation, hypertension, cerebrovascular disease, chronic liver disease, diabetes mellitus, cancer history, chronic renal failure, previous UGIB history), and laboratory data were obtained from the electronic files of the patients.

The Glasgow–Blatchford score (GBS) was calculated using the initial vital and laboratory findings. The endoscopic findings and intervention data were collected from the endoscopic examination reports. The clinical outcomes, including the need for endoscopic treatment, rebleeding, erythrocyte suspension transfusion number, need for embolization/surgery, need for intensive care unit admission, length of hospital stay, and 30-day mortality, were recorded for each patient.

Patients enrolled in the present study were divided into 4 groups: those using anticoagulants, antiplatelets, NSAIDs, and those not using these medications.

Red blood cell replacement was performed to ensure a target hemoglobin (Hb) value of 10 g/dL in patients with cardiac risk factors and 7 to 9 g/dL in other patients. In patients using warfarin, if the patient was unstable, and the international normalized ratio (INR) value was above the therapeutic range, vitamin K, fresh frozen plasma or prothrombin complex concentrate were administered prior to the endoscopic procedure.^[9]^ To evaluate the risk classification of the patients, the initial blood urea nitrogen, Hb, heart rate, systolic blood pressure, signs of melena, syncope, and history of cardiac failure and hepatic disease were determined, and the GBS was calculated.^[10]^

Proton pump inhibitor (PPI) (8 mg/h) was started immediately after the patient admitted to the emergency department and before the EGD examination.^[10]^ Endoscopic treatment was applied to patients in the high-risk group during the endoscopy procedure for Forrest 1a, 1b, 2a, Mallory–Weis, and Dieulafoy lesions.^[11]^ EGD was performed within the first 24 hours of admission. In addition to adrenaline injection during the endoscopic treatment, any of the treatments such as hemoclips, argon plasma coagulation, or Ankaferd blood stopper were applied on the basis of the clinician’s preference. The following factors influenced the clinician’s choice: location-size of the ulcer, the location of the vessel on the ulcer, the position that can be taken with the gastroscope, the position of the clips or coagulation instruments in the bleeding area after they come out of the scope. While adrenaline injection, argon plasma coagulation and hemoclips were generally used, Ankaferd was used as rescue treatment.

After endoscopic treatment, high-dose PPI was given intravenously for 3 days, followed by 2 oral doses of PPI per day.^[10]^ Clinical evidence for recurrent bleeding is defined as the recurrence of hematemesis, development of tachycardia and hypotension after hemodynamic stability, development of melena and/or hematochezia after normal defecation, and a decrease in the Hb value of more than 2 g/dL.^[9]^ For bleeding that could not be stopped after 2 endoscopic treatments, the patient was referred for surgery or embolization.

### 2.1. Statistical analysis

Statistical Package for the Social Sciences (SPSS Inc., Chicago, USA) version 25.0 was used for statistical analysis. Descriptive values are given as the mean ± standard deviation for continuous variables and are presented as numbers and percentages for categorical variables. The chi-square test was used to compare categorical data. The Kruskal ANOVA, and posthoc test analysis was used as a statistical method for the comparison of non-normal and normal distributed numerical variables. A value of *P* < .05 was considered statistically significant.

## 3. Results

The study recruited a total of 297 patients, assigning 32 (10.7%) patients to the NSAID group, 103 (34.6%) patients to the antiplatelet group, 83 (27.9%) patients to the anticoagulant group, and 79 (26.5%) patients to the no drug group. The baseline characteristics, laboratory data, and coexisting diseases of the study’s cohort are detailed in Table 1. The mean age of the study group was 77.07 ± 7.9 years, and 182 (61.3%) of the patients were male. The chi-square test revealed a significant difference in the assessment of gender between the groups (*P* = .001).

**Table 1:** Baseline characteristics of patients.

	NSAİD group n = 32 (10.7%)	Antiplatelet group n = 103 (34.6%)	Anticoagulant group n = 83 (27.9%)	No drug n = 79 (26.5%)	Total N = 297	*P*
Age (years), mean ± SD	77.2 ± 8.5	76.35 ± 7.4	78.48 ± 8.1	76.46 ± 8.1	77 ± 7.9	.269
Male, n (%)	18 (56.3%)	75 (72.8%)	34 (41%)	55 (69.6%)	182 (61%)	**.001**
SBP (mm Hg), mean ± SD	111 ± 18	112 ± 20	107 ± 18	118 ± 25	112 ± 21	**.011**
Hb level on admission, g/dL, mean ± SD	8.1 ± 2.1	8.7 ± 2.3	7.8 ± 2	8.6 ± 2.3	8.3 ± 7.8	**.035**
Platelet, ×10^3^/mm^3^, mean ± SD	260 ± 88	249 ± 94	247 ± 86	241 ± 98	248 ± 92	.811
INR, mean ± SD	1.12 ± 0.2	1.13 ± 0.2	5.17 ± 6.5	1.1 ± 0.1	2.2 ± 3.9	**.001**
Cardiovascular diseases (AF, coronary heart disease, chronic heart failure), n (%)	5 (15.6%)	64 (62.1%)	66 (%79.5)	11 (13.9%)	146 (49%)	**.001**
Hypertension, n (%)	16 (50%)	86 (83.5%)	64 (77.1%)	45 (57%)	211 (71%)	**.001**
Diabetes mellitus, n (%)	4 (12.5%)	31 (30.1%)	17 (20.5%)	11 (13.9%)	63 (21%)	**.031**
Cerebrovascular diseases, n (%)	2 (6.3%)	22 (21.4%)	8 (9.6%)	3 (3.8%)	35 (11%)	**.002**
Chronic liver disease, n (%)	0 (0%)	0 (0%)	0 (0%)	4 (5.1%)	4 (1.3%)	**.011**
Chronic kidney disease, n (%)	2 (6.3%)	19 (18.4%)	8 (9.6%)	9 (11.4%)	38 (12%)	.168
Cancer, n (%)	3 (9.4%)	8 (7.8%)	11 (13.3%)	14 (17.7%)	36 (12%)	.214
Previous episode of UGIB, n (%)	8 (25%)	26 (25.2%)	20 (24.1%)	22 (27.8%)	76 (25%)	.956
Glasgow–Blatchford score, mean ± SD	12.4 ± 3	11.9 ± 3.7	13.3 ± 3.2	11.9 ± 3.6	12.4 ± 3.5	**.047**

Results are expressed as mean ± standard deviation, median (interquartile range), or frequency (%).

Significant *P* values are in bold.

AF = atrial fibrillation, Hb = hemoglobin, INR = international normalized ratio, SD = standard deviation, SBP = systolic blood pressure, UGIB = upper gastrointestinal bleeding.

The initial mean systolic blood pressure differed between groups (*P* = .011). In the group analysis, a statistically significant difference was found between the group using anticoagulants and the group not using medication (*P* = .005).

The Hb level at the time of application was found to differ between groups (*P* = .035), and it was statistically significant when compared to the group using anticoagulants (*P* = .038).

The mean INR value on admission was higher in the anticoagulant group (*P* = .001). The rates of cardiovascular diseases, hypertension, diabetes mellitus, cerebrovascular diseases, and chronic liver diseases differed between groups (*P* = .001, *P* = .001, *P* = .031, *P* = .002, *P* = .011, respectively). The mean Glasgow–Blatchford score was 12.4 ± 3 for the NSAID group, 11.9 ± 3.7 for the antiplatelet group, 13.3 ± 3.2 for the anticoagulant group, and 11.9 ± 3.6 for the no drug group (*P* = .047). Comparisons found a statistically significant difference in the GBS between the groups using anticoagulant and antiaggregant medications (*P* = .01).

Table 2 represents the endoscopic findings of the study groups. The most common endoscopic diagnosis was duodenal ulcer in 92 (31%) patients, followed by gastric ulcer in 74 (24.9%) patients.

**Table 2:** Endoscopic findings of patients.

	NSAİD group n = 32	Antiplatelet group n = 103	Anticoagulant group n = 83	No drug n = 79	Total n = 297
Gastric ulcers	9 (28.1%)	30 (29.1%)	18 (%)	17 (21.5%)	74 (24.9%)
Duodenal ulcer	13 (40.6%)	36 (35%)	16 (%)	27 (34.2%)	92 (31%)
Esophageal ulcer	0 (%)	5 (4.9%)	2 (%)	6 (7.6%)	13 (4.4%)
Mallory–Weiss injury	0 (%)	4 (3.9%)	2 (%)	3 (3.8%)	9 (3%)
Dieulafoy lesion	0 (%)	1 (1%)	1 (%)	1 (1.3%)	3 (1%)
Malignancy	3 (9.4%)	7 (6.8%)	7 (%)	11 (13.9%)	28 (9.4%)
Others	7 (21.9%)	19 (18.4%)	27 (%)	13 (16.5%)	66 (22.2%)
Lesion not visualized	0 (%)	1 (1%)	10 (%)	1 (1.3%)	12 (4.0%)

Table 3 represents the comparison of clinical outcomes of the study groups. One-month mortality was observed in 4 (12.5%) patients in the NSAID group, 11 (10.7%) patients in the antiplatelet group, 5 patients (6%) in the anticoagulant group, and 7 patients (8.9%) in the no drug group. No difference was found between the groups in 30-day mortality (*P* = .634). The mean number of units transfused was 3.75 ± 2.92 in the NSAID group, 3.05 ± 2.97 in the antiplatelet group, 3.73 ± 2.2 in the anticoagulant group, 3.04 ± 2.47 in the no drug group. No difference was found between the groups in number of units transfused (*P* = .183).

**Table 3:** Clinical outcome.

	NSAİD group n = 32	Antiplatelet group n = 103	Anticoagulant group n = 83	No drug n = 79	Total n = 297	*P*
30-day mortality, n (%)	4 (12.5%)	11 (10.7%)	5 (6%)	7 (8.9%)	27 (9%)	.634
Inpatient rebleeding, n (%)	3 (9.4%)	6 (5.8%)	4 (4.8%)	4 (5.1%)	17 (5.7%)	.806
Endoscopic intervention, n (%)	17 (53.1%)	30 (29.1%)	16 (19.3%)	33 (41.8%)	96 (32.3%)	**.001**
Embolization/surgery, n (%)	4 (12.5%)	3 (2.9%)	1 (1.2%)	5 (6.3%)	13 (4.4%)	**.04**
Transfusion required, n (%)	24 (75%)	82 (79.6%)	77 (92.8%)	57 (72.2%)	240 (80%)	**.007**
Number of units transfused, mean ± SD	3.75 ± 2.92	3.05 ± 2.97	3.73 ± 2.2	3.04 ± 2.47	3.31 ± 2.6	.183
Length of stay (days), mean ± SD	6.8 ± 8.2	4.5 ± 4.1	6 ± 4.7	5.4 ± 6	5.44 ± 5.43	.106

Bold values indicate statistically significant values.

The mean length of hospital stay was 6.08 ± 8.2 in the NSAID group, 4.5 ± 4.1 in the antiplatelet group, 6 ± 4.7 in the anticoagulant group, 5.4 ± 6 in the no drug group. No difference was found between the groups in length of hospital stay (*P* = .106). There was an insignificant difference between groups regarding the 30-day mortality, inpatient rebleeding, length of hospital stay (*P* > .05 for all parameters). Notably, the need for transfusion was higher in anticoagulant group (*P* = .007).

In the comparison made regarding the needs for endoscopic treatment, when comparing the NSAID group with the antiplatelet and anticoagulant group, the need for endoscopic treatment was statistically significantly higher in the NSAID group (*P* = .01 and *P* = .001). No significant difference was found between the group using NSAIDs and the group not taking any medication (*P* = .2). No statistically significant difference was found between the antiplatelet group and the anticoagulant group, as well as between the antiplatelet group and the group not using any medication (*P* = .1 and *P* = .07). Statistically significant differences were observed in the necessity for endoscopic procedures between the no drug group and the anticoagulant group. The group not using medication had a greater need for endoscopic intervention (*P* = .002).

## 4. Discussion

UGIB is a common cause for hospital admission in older adult patients, and it is associated with high morbidity and mortality if not adequately managed. Despite the increase in endoscopic treatment modalities, it still remains an important health problem. Risk factors include *H. pylori* infection, higher age, smoking, medication, and history of liver disease.^[12]^ As life expectancy increases and comorbid diseases occur, drug use increases, and as a result, gastrointestinal bleeding becomes more common. In this study, an insignificant difference was detected between groups in terms of the one-month mortality, rebleeding, transfusion amount, and hospital stay duration. Although antithrombotics and NSAIDs are known to increase the frequency of bleeding, it has been reported that they do not cause any significant clinical change in studies conducted on the elderly patient population.^[13]^ When the group not using medication was evaluated as a control for the need for endoscopic treatment, no difference was found with NSAIDs and antiplatelet agents, while a lower rate was observed in those using anticoagulants.

Peptic ulcer bleeding can lead to serious consequences in the elderly, with a mortality rate of 5% to 10%.^[14]^ In a study including patients over 70 years of age, the 30-day mortality rate was found to be 7.8% in patients admitted due to upper GI bleeding.^[15]^ In another study involving people over the age of 74, the mortality rate was found to be 11.8%.^[16]^ In a study published by Ortiz et al, with patients over 18 years of age, a mortality rate of 4.7% was observed in those taking anticoagulants, 5.6% in those taking antiplatelet drugs, and 5.8% in the patient group not using medication.^[17]^ Despite therapeutic developments in GI bleeding, a mortality rate of 5% to 10% can be observed.^[18]^ In our study, the 30-day mortality rate was found to be 9.0%. The high mortality rates may be due to the advanced age of the patients and the accompanying diseases. Medication usage did not significantly change the mortality. The increase in average life expectancy, high average age of the patient group, and the fragility of patients due to multiple comorbid conditions may have played a role in the mortality rates remaining unchanged over the years. However, further prospective studies are warranted in the evaluation of mortality rates in geriatric population that assess the factors mentioned above.

Concomitant cardiovascular disease, cerebrovascular diseases, diabetus mellitus, and hypertension were detected more frequently in patients with antiplatelet and anticoagulant use. The presence of comorbid conditions in patients using antiplatelet and anticoagulant medications was an expected outcome. In a study including 2075 patients, the most common comorbidities in patients admitted due to UGIB were hypertension (32.7%) and diabetes mellitus (30.4%), respectively.^[19]^ In our present study, the most common comorbidity was hypertension in all groups. In total, 70% of the patients had hypertension. Since our study included a geriatric patient group, the rate of hypertension may have been higher.

Early endoscopy (≤24 hours from the time of patient presentation) is associated with lower in-hospital mortality, shorter length of stay, and lower total hospital costs, and should be performed in patients with acute UGIB.^[20]^ Hemodynamic stability must be achieved before early endoscopy. The prognosis has been found to be worse in patients who underwent the procedure without achieving patient stabilization. In our study, all patients were evaluated by endoscopy within 24 hours as recommended by the guidelines.

In a study published by Yamaguchi et al, the Hb value at the time of admission (8 vs 8.9 g/dL) and the number of replacements were found to be lower in the medication-using group.^[21]^ In our study, the number of replacements were similar between groups. The average replacement number across all groups was 3.3 units. No significant difference was observed in Hb levels between the group that did not take medication and those using it when comparing measure at the time of application. However, the number of patients that required transfusion differed between the groups. The difference in the number of patients requiring transfusion between the groups may be explained by the higher target Hb value due to comorbid cardiovascular diseases in the medication group.

One of the most frequently used scoring systems to determine the need for endoscopic treatment and mortality in patients presenting with acute UGIB is the Glasgow–Blatchford Scoring system.^[10,22]^ The patient’s need for endoscopic treatment increases with an increasing score. Zero points represent a low-risk group, and mortality and emergency endoscopic need rates are low. Although age is not a determining factor in scoring in the GBS, the scoring is greater than zero in most patients due to other variables.^[23]^ In our study, groups receiving NSAIDs, antiplatelet agents, and anticoagulants were compared one by one with those not using any medication in terms of GBS, and no significant differences were found among them. In the analysis of the groups, only it was significantly higher in patients using only anticoagulants compared to antiplatelet therapy.

The most common cause of non-variceal UGIB is peptic ulcer, with a rate of approximately 35% to 50%.^[24]^ Peptic ulcer bleeding is the most common cause of UGIB in the elderly, too.^[25]^ In our study, duodenal ulcer was the most common cause of UGIB in both groups of geriatric patients.

Endoscopic treatment was also applied to patients with Forrest 1a, 1b, or 2a,^[26]^ those diagnosed with Mallory–Weis, and patients with Dieulafoy lesions. The fact that patients using anticoagulants require less endoscopic treatment compared to those using NSAIDs and no drug group may be related to the normalization of high INR levels in the anticoagulant patients group, which is associated with the spontaneous cessation of bleeding. In a study conducted with 504 geriatric patients in Japan in 2017, the rate was found to be 46%.^[27]^ In our study, an endoscopic procedure was performed at a higher rate of 67%.

When endoscopic treatment is inadequate in gastrointestinal bleeding, surgical or radiological treatment may be required. In a study conducted in Finland with 282 patients over the age of 70, the proportion needing surgery was found to be 2.1%. In this study, patients with a previous history of UGIB were excluded from the study.^[15]^ There are publications reporting the need for surgery in 5.4% to 8.7% of patients using anticoagulants.^[11,28]^ In our study, the surgery/embolization rate was 4.4%, which was consistent with the literature. Drug use did not increase the need for surgery or embolization.

In a study conducted with a geriatric patient group from Turkey, the average hospital stay due to UGIB was found to be 7.8 days, and the average number of replacements was 2.7 units.^[29]^ The average number of replacements in patients over 70 years of age with UGIB was found to be 4.6 units.^[15]^ In the study conducted by Solakoğlu et al with a patient group over the age of 18, the average length of stay was found to be 3.4 days in patients using antiplatelets, anticoagulants, and NSAIDs and 3.2 days in those who did not use these drugs.^[11]^ In a study conducted by John et al, which included 104 patients, it was shown that antiplatelet treatments did not change the length of hospital stay and the amount of red blood cells replaced.^[17]^ In our study, the number of replacements (3.3 units) and length of hospital (5.4 day) stay were found to be similar to previous studies. Using medication does not seem to affect the length of hospital stay.

The limiting aspects of our study are as follows: It was conducted retrospectively in a single center with a limited number of patients; patients who could not undergo gastroscopy due to a poor general condition, patients who were hospitalized for a reason other than bleeding, and patients who had UGIB during their hospitalization were not included in the study. Alcohol, PPI use (which may affect bleeding status), and the presence of *H. pylori* in patients were not investigated. In addition, it is difficult to evaluate whether the cause of mortality in the very elderly occurs solely due to the bleeding or develops secondary to the deterioration of the general condition of the patient.

## 5. Conclusions

Antiplatelets, anticoagulants, and NSAIDs are frequently used in geriatric patients who present with gastrointestinal bleeding, due to the high rate of concomitant comorbidities. However, we demonstrated that NSAID, antiplatelet, or anticoagulant use was not associated with adverse outcomes in geriatric patients who presented with UGIB. Further prospective studies with larger sample size are needed to evaluate the role of these drug groups on the clinical outcomes of UGIB.

## Author contributions

**Conceptualization:** Soner Onem.

**Data curation:** Soner Onem, Muge Gul Gulecoglu Onem.

**Formal analysis:** Soner Onem, Süleyman Dolu.

**Investigation:** Süleyman Dolu.

**Methodology:** Soner Onem, İbrahim Ethem Güven.

**Resources:** Muge Gul Gulecoglu Onem.

**Software:** İbrahim Ethem Güven.

**Supervision:** Muge Gul Gulecoglu Onem, Taner Akyol.

**Validation:** İbrahim Ethem Güven.

**Visualization:** Süleyman Dolu, Taner Akyol.

**Writing – original draft:** Soner Onem.

**Writing – review & editing:** Soner Onem.
